# Unveiling pedagogical effects of blended multimedia and slide-based approaches on students’ self-efficacy and performance in over-the-counter medication counseling: an explanatory mixed methods study

**DOI:** 10.1186/s12909-025-08094-7

**Published:** 2025-10-31

**Authors:** Yen-Ming Huang, Fang-Ju Lin, Yao-Hsing Wang, Olayinka Shiyanbola, Hsun-Yu Chan, Yunn-Fang Ho

**Affiliations:** 1 School of Pharmacy, College of Medicine, National Taiwan University, Taipei City, 100025 Taiwan; 2 Graduate Institute of Clinical Pharmacy, College of Medicine, National Taiwan University, Taipei City, 100025 Taiwan; 3 Department of Pharmacy, National Taiwan University Hospital, Taipei City, 100229 Taiwan; 4 Honest Pharmacy, Taichung City, 408483 Taiwan; 5 Department of Clinical Pharmacy, College of Pharmacy, University of Michigan, Ann Arbor, MI 48109 USA; 6 Department of Industrial Education, National Taiwan Normal University, Taipei City, 106308 Taiwan

**Keywords:** Communication, Counseling, Education, Multimedia, Over-the-counter, Pedagogy, Pharmacy

## Abstract

**Background:**

Addressing healthcare communication skills through a multimedia approach may enrich undergraduate pharmacy students’ medication counseling competence. This study explored the impacts of a blended classroom teaching approach (incorporating newly designed video-based materials into conventional slide-based lectures) on students’ self-efficacy and performance in over-the-counter (OTC) medication counseling services.

**Methods:**

Using a sequential explanatory mixed methods approach, two cohorts of undergraduate pharmacy students enrolled in the 16-week *Introduction to Community Pharmacy* course were investigated. The cohort expected to graduate in 2025 served as the control group and received 4 weeks of conventional instruction using PowerPoint slides to introduce professional communication and counseling skills. In contrast, the intervention cohort expected to graduate in 2026 received 3 weeks of PowerPoint-based instruction, followed by an additional week of video-rich learning in OTC counseling. Pre- and post-course questionnaires were administered to assess students’ self-efficacy in OTC counseling. Moreover, their counseling performance was evaluated through role-playing exercises with standardized patients during the final week of the course. Multivariate regression analyses determined the effectiveness of each teaching approach in enhancing self-efficacy and counseling performance. Semi-structured interviews were conducted and analyzed using inductive thematic analysis to gain insights into students’ perspectives and experiences with the two teaching approaches.

**Results:**

Both cohorts exhibited enhanced self-efficacy in medication counseling through the two approaches. However, the group that received the blended multimedia approach showed significantly greater improvement in counseling performance than its counterpart (*p* = 0.007). Students in the intervention cohort appreciated how video-based instruction provided visual and auditory cues, helping them grasp the structured flow of OTC counseling. Witnessing real-case scenarios in videos facilitated their understanding and appreciation of counseling techniques in real-life pharmacy settings.

**Conclusion:**

The study highlights that integrating multimedia instruction, particularly videos, significantly improved pharmacy students’ OTC counseling performance, while self-efficacy showed improvement without a significant difference compared to traditional slide-based lectures. To better support the development of counseling competence, a blended instructional approach that combines video-based learning with conventional lectures is recommended.

## Introduction

Over-the-counter (OTC) medications provide the public with easy access to managing ailments without a prescription; however, inappropriate use of OTCs can pose risks to individual health [1]. Improper self-diagnosis, incorrect dosage, prolonged misuse, adverse drug reactions (ADRs), and potential drug interactions are among the risks associated with inappropriate OTC use [2]. Previous research observed one-third to one-half of ADRs resulting from self-medications were attributable to OTCs [3]. Moreover, 6% of ADR presentations to emergency departments were caused by OTC medications [4]. Misunderstanding of the dosing instructions on medication labels has been identified as a root cause of these unexpected ADRs [5, 6]. Pharmacists play a critical role as the first and last point of contact in the provision of OTCs [7], assisting clients in navigating the self-medication journey for managing ailments through symptoms evaluation, product selection, proper medication use, and ADR prevention [8].

Medication safety and overall well-being can be enhanced by pharmacy services, which rely on effective patient-pharmacist communication [9]. This underscores the importance of pharmacists proactively engaging patients in medication counseling to ensure safe, appropriate, and effective use of self-medication [10]. Nevertheless, Kim et al. indicated that pharmacists’ counseling skills of self-medication could be improved in community pharmacies [11]. Suboptimal consultation performance in community pharmacies often stems from inadequate information gathering or advice provision, as conventional patient-pharmacist communication has been unidirectional, with a focus on information provision, leaving patients’ voices unheard [12]. Effective gathering of relevant patient backgrounds and providing appropriate information are crucial for addressing patient conditions and selecting suitable therapies [13]. Given the potential risks associated with self-medication and the growing need for up-to-date counseling competencies, Kerr et al. advocated for the inclusion of comprehensive training in medication counseling as part of pharmacy essential curricula [14]. Such training should incorporate dynamic communication skills that consider various context-dependent components (e.g., patient characteristics, the nature of the medication or condition, patient emotions or attitudes, and the time and resources available), thereby empowering students to adopt patient-centered communication styles [15]. By doing so, pharmacy students will be better equipped to meet patient needs, promote safer medication use, and enhance healthcare outcomes.

Drawing on the Social Cognitive Theory (SCT), it is proposed that learning occurs through observing and imitating others’ actions [16]. Digital technology in education holds great potential for creating multimedia learning environments by combining text, sounds, and images [17]. Mayer has emphasized the significance of video as one of the multimedia learning approaches to maximizing the use of learners’ cognitive infrastructure when aligned with the SCT principles [18, 19]. Previous research suggests that videos, combining audio and visual elements, enhance recall compared to presentations that rely solely on visuals or audios [20]. Medication counseling, given its complex and dynamic nature within the healthcare journey, benefits from incorporating videos into educational materials. This integration allows multiple symbol systems to represent intricate and evolving social contexts and events, thereby assisting students in constructing rich and dynamic mental models of learning [20]. Consequently, this approach is likely to activate relevant prior knowledge that students can apply to better problem-solving [20].

While the Multimedia Learning Theory (MLT) in cognitive psychology suggests that learning outcomes improve when visual and auditory information are properly co-presented [21], many pharmacy courses in Taiwan still adhere to a traditional teaching format. These courses typically include lectures accompanied by text-rich PowerPoint slides, even in practical skill-based courses, such as communication. The issue with relying solely on one-way, lecture-based instruction without using multimedia resources or hands-on practice is that it may pose challenges for students in processing procedural knowledge [22].

In this study, we devised a teaching program using a blended multimedia learning strategy to enhance pharmacy students’ competence in medication counseling. We hypothesized that incorporating captioned videos depicting real-life scenarios, alongside conventional in-class lectures, would improve self-efficacy and performance of pharmacy students in the context of OTC counseling. Self-efficacy is a crucial factor that influences student performance [23]. It refers to a learner’s belief in their ability to successfully complete specific tasks or navigate particular learning situations [24]. In pharmacy education, especially in skill-based activities such as medication counseling, self-efficacy reflects students’ confidence in their ability to communicate effectively with patients, apply pharmacological knowledge, and make sound clinical decisions. Those with strong self-efficacy tend to attribute their outstanding performance to their increased dedication to professional growth, consistent engagement in active learning, and steadfast perseverance in healthcare practice [25]. We aimed to investigate whether and in what ways the blended multimedia teaching approach can positively impact learners’ communication self-efficacy and counseling performance.

## Methods

**Fig. 1:**
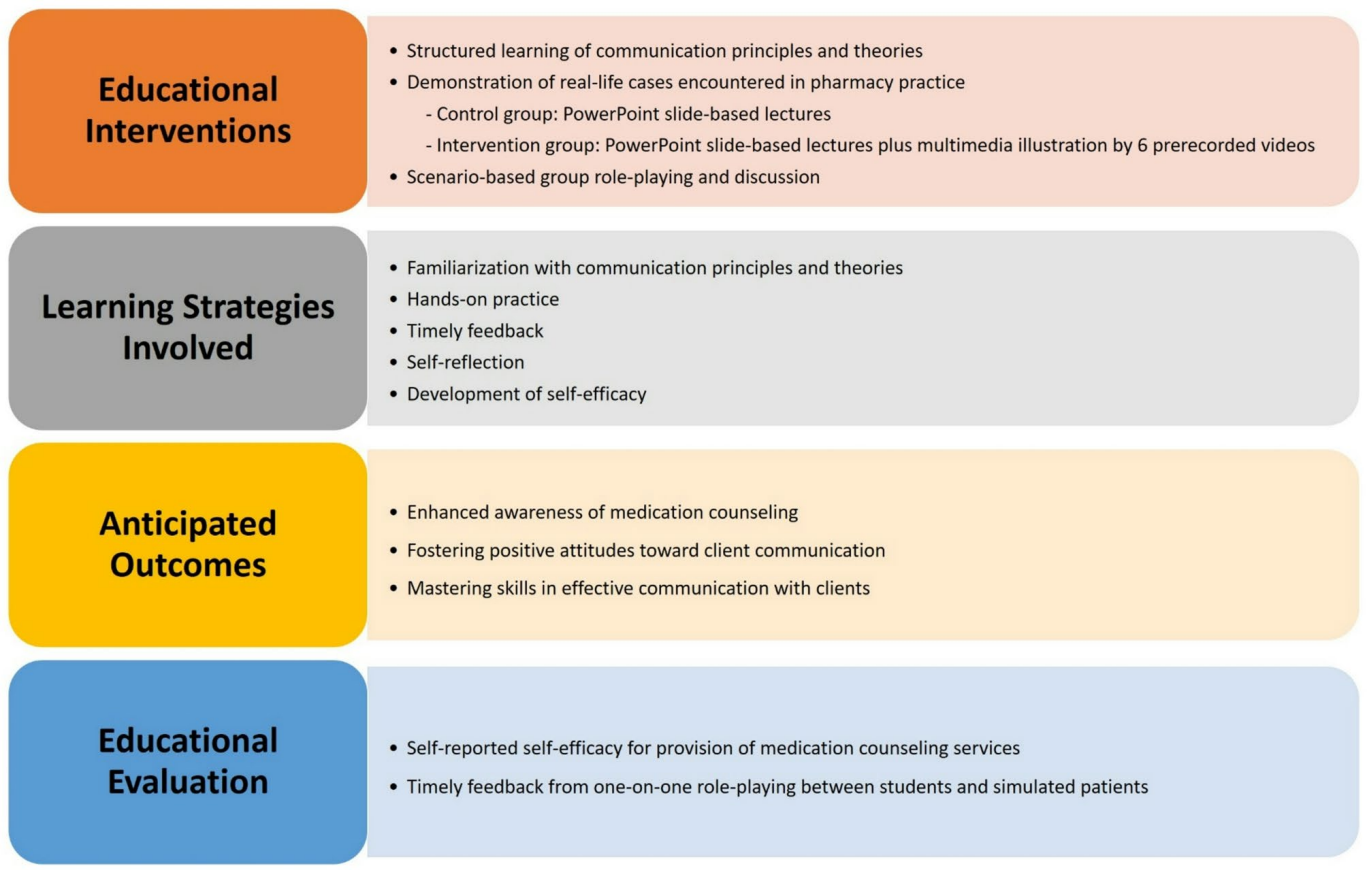
Conceptual framework of course design, learning strategies, anticipated outcomes, and evaluation methods

This study involved two cohorts of third-year undergraduate pharmacy students, aiming to assess and compare the impact of two pedagogical approaches (i.e., the conventional slide-based approach versus the blended multimedia approach) on communication self-efficacy and counseling performance of OTCs. Data were collected through a structured survey, a performance checklist, and semi-structured interviews. Figure 1 illustrates the conceptual framework of course design, learning strategies, anticipated outcomes, and evaluation methods of the study.

### Study design

This study used an explanatory sequential mixed methods approach [26], as illustrated in Appendix 1. This approach began with a quantitative phase followed by a subsequent qualitative phase [27]. Initially, quantitative data were collected and analyzed through a self-report survey and a performance checklist to assess the impact of different pedagogical approaches on student communication self-efficacy and OTC counseling performance. The subsequent qualitative phase involved one-on-one interviews as a follow-up to the survey results. These interviews delved deeper into understanding how the two teaching approaches influenced students’ performance in OTC counseling. Ultimately, the study integrated the findings from both quantitative and qualitative phases to explore how they complemented each other. This mixed methods approach allowed researchers to gain a more comprehensive perspective by combining statistical results with participants’ insights [28]. Throughout both phases, data collection occurred via face-to-face interactions between February 2022 and June 2023.

### Course design and intervention

The two consecutive cohorts, expected to graduate in 2025 and 2026, were in the third year of the 6-year pharmacy curriculum at the National Taiwan University. These cohorts were enrolled in the *Introduction to Community Pharmacy* course, with the Class of 2025 serving as the control cohort and the Class of 2026 as the intervention cohort. The *Introduction to Community Pharmacy* is a 16-week, 2-credit course mandatory for all pharmacy majors [29]. This study specifically centered on the 4-week session on medication counseling and communication in pharmacy practice, with weekly two-hour sessions.

The primary distinction in pedagogical approaches between the two cohorts was in the manner of delivering the Self-Assessment-Informed Decision-making-System (SAIDS) approach to OTC counseling [10]. The SAIDS approach has been used in community pharmacy practice and demonstrated to effectively enhance consumers’ understanding of proper OTC use by entry-level pharmacists [10]. This structured approach comprises five counseling steps to identify and address patient needs, including uncovering symptoms and OTC needs, inquiring about allergies and medication histories, reaffirming medication indications, directing correct medication use, and emphasizing strategies for self-care empowerment [10]. Except for incorporating video-based learning, the remaining teaching materials were consistent across both cohorts. Table 1 depicts the course content provided to both cohorts. All instructional resources were made available through NTU COOL, the university’s web-based learning management system, which allows students to conveniently access and review materials. NTU COOL serves as a centralized digital repository where instructors upload various educational materials, including lecture slides, reference links, videos, and other media, to support student learning and allow for flexible and on-demand access.

**Table 1:** Comparison of pedagogical elements between conventional and blended multimedia approaches

	Pedagogical approach and contents	Time (min)	Class of 2025 (conventional)	Class of 2026 (blended multimedia)
Week 1: Course introduction	• Pre-survey (self-efficacy)	15	X	X
Week 2–3: Progress and current practices in community pharmacy in Taiwan				
Week 4: Basics for communication and interpersonal relationships	• Power-point lecturing on: (1) Definition and importance of communication (2) Strategies for effective communication with the general public and healthcare professionals	50	X	X
	• Case practice: Students demonstrate how to communicate with different audiences about medication counseling through group discussion and exercises	50	X	X
Week 5: Identification of individual needs and differences for provision of tailored medication counselling and	• Power-point lecturing on: (1) Cultural and life experience link to medication use (2) Evaluation of individual differences in medication use	50	X	X
	• Group discussion on the way to provide tailored pharmaceutical care based on individual needs	50	X	X
Week 6: Application of individualized medication counselling in pharmacy practice	• PowerPoint lecturing on: (1) Exploration of common questions from community residents (2) Provision of appropriate responses to answer questions in the community setting	100	X	X
Week 7: Orientation of the SAIDS approach to OTC counselling and group practice	• PowerPoint lecturing on: Introduction and application of the SAIDS approach to OTC counseling a. **S**urfacing **S**ymptoms and OTC needs b. Inquiring about **A**llergy and medication histories c. Reaffirming medication **I**ndication d. **D**irecting correct medication use e. Reiterating **S**trategies to empower **S**elf-care and to cope with common **S**ide effects	50	X	
	• Video-based discussion on: (1) Introduction and application of the SAIDS approach to OTC counseling (PowerPoint) (2) Utilization of 6 pre-recorded cases to link the SAIDS approach to pharmacy practice (3) Discussion on how to map the SAIDS principles to the video cases	50		X
	• Role-playing practice: Students employ the SAIDS approach to OTC counseling for the assigned case through group discussion and exercises	50	X	X
Week 8–15: Medication, personnel, and financial management in community pharmacies; overview of the long-term healthcare system; the role of pharmacies from a public health perspective, legal issues and case examples related to community pharmacy practice				
Week 16 (Final)	• Post-survey (self-efficacy)	15	X	X
	• Evaluation of role-playing in OTC counseling (counseling skills)	15	X	X

For the control cohort (Class 2025), the 4-week session started with a 3-week introduction to professional communication skills, with a specific focus on OTC medication counseling. Students were taught using a conventional approach, with the content being delivered through in-class lectures. The lectures covered a range of topics, including understanding communication basics, tailoring medication counseling to client needs and differences, and the application of individualized medication counseling in pharmacy practice. Most of these materials were presented through PowerPoint slides featuring plain text and static pictures. In the fourth week, the instructor introduced the SAIDS approach for OTC counseling through PowerPoint slides. Afterwards, students were provided with 6 case scenarios to engage in hands-on practice, involving group discussions and in-class role-playing exercises. The scenarios included various dosage forms (e.g., oral and topical) and OTC therapeutic classes (e.g., analgesics and gastrointestinal products). Students were tasked with responsibilities, such as recommending suitable OTCs, aiding patients in correct OTC usage, recognizing patients with OTC misconceptions, and dispelling these misconceptions.

Students in the intervention cohort (Class 2026) received a modified approach. In addition to the conventional teaching approach used for the control cohort, they had the opportunity to acquire counseling skills through multimedia-inspired learning materials at their own pace. This encompassed viewing scenario-based videos illustrating real-life OTC counseling situations, engaging in group role-playing exercises, and watching pre-recorded videos that demonstrated how to apply the SAIDS approach in OTC counseling. These videos, each lasting 3–5 min, were designed based on the 6 scenarios presented to the conventional cohort. The counseling process was verbally presented and captioned for clarity in each video, demonstrated by simulated patients and a practicing pharmacist to showcase patient-pharmacist interactions. In-class discussions were facilitated to explain and link SAIDS approach to the effective delivery of OTC counseling.

### Sampling and recruitment

Convenience sampling was used to streamline the participant recruitment process, where students enrolled in the 2022 and 2023 *Introduction to Community Pharmacy* courses were invited during the designated semesters. Eligible students received information sheets and were obligated to sign informed consent before becoming study participants. Since the outcome measures were part of the mandatory course evaluation, students who did not provide informed consent were still required to complete the evaluation. Nevertheless, their data were excluded from the subsequent analyses. Ethical approval for all study procedures was obtained from the Research Ethics Committee at the National Taiwan University Hospital (202109017RIND).

To ensure sufficient statistical power, we conducted a priori power analysis for a multiple regression test involving five predictors using G*Power 3.1 with power of 0.80 and two-tailed α = 0.05 [30]. A sample size of 92 participants would be needed to detect a medium-sized effect (*f*^2^ = 0.15), and a larger sample size would further enhance the ability to detect the intervention effect [31].

### Evaluation methods

#### Quantitative measures

Each participant in both the intervention and control groups were required to completed a 14-item pre-to-post survey, which assessed their self-efficacy in OTC counseling [32, 33]. This self-report survey was administered during the first week of the semester and again one week prior to the final (Table 1). Using a 5-point Likert scale (ranging from 1 = not confident at all to 5 = highly confident), students were asked to indicate their confidence level in performing OTC counseling, including patient evaluation and information delivery. The item scores were aggregated to calculate a total self-efficacy score, ranging from 14 to 70, with a higher score indicating a greater confidence level in effectively addressing the crucial stages of medication counseling [29].

As part of the final summative assessment, students’ proficiency in providing OTC counseling was evaluated using a 20-item checklist (Appendix 2) sourced from relevant literature [32, 34, 35]. This evaluation specifically focused on scenario-based assessment of OTC counseling. Each item in the checklist corresponded to a specific element that should be demonstrated during counseling practice and was measured using a binary response option (yes/no). In the final course assessment, each student participated in a scenario-based evaluation by engaging in one-on-one OTC counseling with a simulated patient. The evaluation lasted for approximately 10–15 min, during which students were encouraged to tailor their counseling approach to meet the patient’s needs, following the SAIDS principle. Eight trained pharmacist evaluators, acting as simulated patients, provided tailored counseling feedback immediately after the evaluation. These evaluators verified if the students’ performance met the respective checklist items. Students were awarded one point for each criterion met, and a higher total score indicated a greater level of performance in applying the SAIDS approach in the simulated scenario [29].

#### Qualitative methods

To acquire a more comprehensive insight into the impacts of the course, an interview guide (Appendix 3) was developed and used to conduct semi-structured interviews with selected interviewees. The interviewees involved five students from each cohort who had completed the pre- and post-course surveys. Although there are no strict rules regarding sample size in qualitative interviews, a sample of 9 to 17 participants is generally considered sufficient for a thematic analysis approach [36]. Purposive sampling was used to recruit students at the end of the course, ensuring diverse perspectives based on gender, changes in self-efficacy scores, and performance in the counseling role-play. Invitations were sent via email, and interested students were screened to achieve variation across these characteristics. Interviews were conducted by a member of the research team in a private office on the National Taiwan University campus and lasted between 30 and 60 min. All interviews were audio-recorded and transcribed verbatim to ensure accuracy for subsequent analysis.

### Analysis

#### Quantitative data

The characteristics of the study participants and their responses were summarized using frequency distributions and descriptive statistics. To examine the difference in mean total self-efficacy scores before and after the intervention within each cohort, paired t-tests were conducted. Furthermore, an independent t-test was performed to compare the differences in mean total self-efficacy scores between the two cohorts. A linear regression model was employed to compare the differences in mean total scores of the counseling performance between the two cohorts, while also identifying factors that might predict students’ counseling performance, after controlling for associated covariates (i.e., sex, age, pre-class self-efficacy score, post-class self-efficacy score, and the pedagogical approach). All statistical analyses were performed using SPSS version 28. A p-value of less than 0.05 was considered statistically significant, and all tests were two tailed.

#### Qualitative data

Inductive thematic analysis was employed to analyze the interview transcripts. By adopting an inductive approach, researchers are not constrained by pre-existing theoretical frameworks or preconceptions during data analysis [37]. This method provides flexibility and allows for a comprehensive understanding of the entire dataset [38]. Initially, the researchers assigned data segments to generate main codes, employing both descriptive coding and in vivo coding techniques [39]. Subsequently, pattern coding was utilized to group these units into a smaller number of categories or themes that related to the research questions [40]. Investigator triangulation was applied to establish an overall coding taxonomy and ensure the credibility of the findings. Two researchers independently coded all transcripts and reached a consensus on each code and its interpretation, thereby grounding the findings in the text [41]. MAXQDA 2020 software facilitated data management through organizing and categorizing the identified themes.

### Integration of the quantitative and the qualitative data sets

Integration occurred at three levels, covering the design, methods, and interpretation levels [42]. An explanatory sequential design was carried out to implement integration at the design level, where the interview questions for the qualitative data collection were based on the survey results [27]. At the methods level, the quantitative and qualitative phases were connected through sampling, whereby the participants for the qualitative study were based on the survey scores obtained in the quantitative phase. We used a weaving approach at the interpretation level to map qualitative findings to quantitative findings on a concept-by-concept basis [43].

## Results

### Quantitative findings

Of the 100 students who consented to participate in the research, 97 (97.0%) completed the survey. They had an average age of 21.2 (SD = 1.5) years. Among the two cohorts, females (*n* = 56) accounted for 57.7% of the study sample. Specifically, 50 students were taught by a slide-based approach for medication counseling, while 47 students were instructed using a blended multimedia approach.

Table 2 presents a comparison of students’ self-efficacy scores in OTC counseling before and after the course, across two groups that received different pedagogical approaches. In the slide-based instruction group, the average baseline self-efficacy score was 39.02 ± 11.18, which increased significantly to 49.96 ± 6.02 after the 4-week course (*p* < 0.001). Similarly, the group taught using a blended multimedia approach showed a significant improvement, with scores rising from 36.07 ± 12.82 to 50.60 ± 6.14 (*p* < 0.001). Both instructional approaches resulted in significant improvements in students’ self-efficacy: 10.93 ± 9.35 in the slide-based group (*p* < 0.001) and 14.52 ± 10.69 in the blended multimedia group (*p* < 0.001). However, the difference in the magnitude of improvement between the two cohorts was not statistically significant (*p* = 0.098).

**Table 2:** Pre-to-post comparison of communication self-efficacy score within two study cohorts

Item	Question	Cohort 2025 (conventional; *n* = 50)				Cohort 2026 (blended multimedia; *n* = 47)			
		Pre-class^a^	Post-class^a^	Pre-post difference^a^	p-value	Pre-class^a^	Post-class^a^	Pre-post difference^a^	p-value
1.	I can accurately identify a patient’s problem.	3.11 (1.09)	3.64 (0.53)	0.53 (0.94)	< 0.001	3.05 (1.10)	3.57 (0.70)	0.52 (1.07)	0.003
2.	I can consistently ask about a patient’s medical conditions.	3.18 (0.96)	3.84 (0.56)	0.67 (0.93)	< 0.001	3.02 (1.18)	3.76 (0.69)	0.74 (1.11)	< 0.001
3.	I can correctly determine whether a patient can self-treat with an over-the-counter (OTC) product(s).	2.73 (0.94)	3.56 (0.59)	0.82 (1.01)	< 0.001	2.60 (1.13)	3.69 (0.64)	1.10 (1.01)	< 0.001
4.	I can recommend OTC product(s) that will address all of a patient’s symptom(s).	2.53 (0.97)	3.36 (0.74)	0.82 (1.03)	< 0.001	2.52 (1.07)	3.31 (0.56)	0.79 (1.07)	< 0.001
5.	I can accurately counsel a patient on an OTC product.	2.64 (0.98)	3.56 (0.59)	0.91 (1.08)	< 0.001	2.38 (0.96)	3.48 (0.59)	1.10 (0.93)	< 0.001
6.	I can accurately counsel a patient on what a specific OTC product is for.	2.89 (1.13)	3.89 (0.68)	1.00 (1.15)	< 0.001	2.62 (1.04)	3.83 (0.66)	1.21 (0.95)	< 0.001
7.	I can accurately counsel a patient on how to take a specific OTC product.	3.13 (1.08)	4.02 (0.58)	0.89 (1.01)	< 0.001	2.93 (1.11)	3.95 (0.58)	1.02 (1.02)	< 0.001
8.	I can accurately counsel a patient on when he/she should start seeing a benefit from a specific OTC product.	2.71 (0.94)	3.38 (0.75)	0.67 (0.98)	< 0.001	2.43 (0.97)	3.40 (0.77)	0.98 (0.98)	< 0.001
9.	I can accurately counsel a patient on common adverse effects that may occur with a specific OTC product.	2.93 (1.03)	3.78 (0.82)	0.82 (0.94)	< 0.001	2.83 (1.08)	3.83 (0.70)	1.00 (1.04)	< 0.001
10.	I can accurately counsel a patient on when to follow up with a physician after recommending an OTC product.	3.02 (1.03)	3.78 (0.82)	0.76 (0.91)	< 0.001	2.81 (1.23)	4.07 (0.75)	1.26 (1.13)	< 0.001
11.	I can identify drug allergies that affect product selection for a self-care patient.	2.49 (0.90)	3.27 (0.69)	0.78 (0.90)	< 0.001	2.19 (1.07)	3.40 (0.67)	1.21 (0.98)	< 0.001
12.	I can identify medications a patient may be taking that affect product selection for a self-care patient.	2.47 (0.94)	3.24 (0.61)	0.78 (0.82)	< 0.001	2.24 (1.01)	3.29 (0.74)	1.05 (0.99)	< 0.001
13.	I can identify concomitant disease states that affect product selection for a self-care patient.	2.49 (0.92)	3.13 (0.55)	0.64 (0.83)	< 0.001	2.07 (0.97)	3.21 (0.72)	1.14 (1.03)	< 0.001
14.	I can describe patient specific factors that influence the selection of the dosage form of an OTC product.	2.69 (0.97)	3.53 (0.66)	0.84 (0.93)	< 0.001	2.38 (1.13)	3.79 (0.78)	1.41 (1.29)	< 0.001
Total		39.02 (11.18)	49.96 (6.02)	10.93 (9.35)	< 0.001	36.07 (12.82)	50.60 (6.14)	14.53 (10.69)	< 0.001

^a^Data were expressed as mean and standard deviation of the survey items

In comparison to students who were exposed to the slide-based approach, those who received the blended multimedia approach demonstrated a higher average total score in OTC counseling performance (slide-based: 13.09 ± 2.65, blended multimedia: 14.69 ± 2.78; *p* = 0.007). Specifically, students who were instructed the blended multimedia approach tended to excel in courtesy (*p* < 0.001), nonverbal communication (*p* < 0.001), and the use of multiple communication strategies (*p* = 0.005) when compared to their counterparts (Table 3). Furthermore, students who learned counseling through the blended multimedia approach (β = 0.332; *p* = 0.005) and who reported higher self-efficacy scores after the class (β = 0.318, *p* = 0.002) were associated with enhanced performance in providing OTC counseling (Table 4).

**Table 3:** Comparison of scores for counseling skills between cohorts

Construct	Cohort 2025^a^ (conventional; *n* = 50)	Cohort 2026^a^ (blended-multimedia; *n* = 47)	*p*-value
Courtesy	1.40 (0.62)	1.93 (0.26)	< 0.001
Needs assessment	5.27 (1.03)	5.26 (1.27)	0.985
Medication recommendation	4.20 (1.10)	4.67 (1.10)	0.051
Response confirmation	1.33 (0.74)	1.21 (0.78)	0.467
Nonverbal communication	0.22 (0.42)	0.62 (0.49)	< 0.001
Empathy expression	0.51 (0.51)	0.57 (0.50)	0.578
Use of multiple communication strategies	0.16 (0.37)	0.43 (0.50)	0.005
Total	13.09 (2.65)	14.69 (2.78)	0.007

^a^Data were expressed as mean and standard deviation of the survey items

**Table 4:** Regression model of associated factors that predict students’ competence in communication skills for over-the-counter medication counseling (*n* = 97)

Dependent variable: Total score of students’ communication skills for over-the-counter medication counseling				
	B	SE	β	p value
Post-class self-efficacy score	0.155	0.054	0.332	0.005
Receiving slide-based and video-based materials^a^	1.783	0.565	0.318	0.002
Age	−0.309	0.196	−0.166	0.120
Pre-class self-efficacy score	0.011	0.027	0.047	0.689
Male student^b^	−0.157	0.556	−0.028	0.778
*F*	5.988^***^			
*Adjusted R* ^2^	22.5%			

*B *Unstandardized coefficient, *SE* Standard error, *β S*tandardized coefficient

^***^P < 0.001

^a^ Compared with those received slide-based materials

^b^ Compare with female students

### Qualitative findings

Ten interviewees were recruited for qualitative interviews, with an even distribution of 5 individuals from each study cohort. The sample consisted of 6 females (60.0%) and 4 males (40.0%), aged 20–23 years. We present the participants’ viewpoints and quotes on how the SAIDS approach and various ways of delivering SAIDS principles had influenced students’ learning in OTC counseling.

#### The SAIDS approach presents a structured framework for progressively learning OTC counseling

All students highlighted the SAIDS approach as a structured counseling model that aided in identifying patients’ needs and suggesting appropriate products or solutions to alleviate their discomfort. Some noted that the 5-step approach guided them systematically through facilitating shared-decision making in OTC self-care by prompting pertinent questions, assessing patient needs, and delivering relevant information. The first two steps (i.e., surfacing symptoms and inquiring about allergies) helped distinguish whether OTC self-care is suitable or if a medical referral is warranted based on patients’ ailment presentations. The last three steps (i.e., reaffirming OTC indications, directing OTC use, and reiterating self-care strategies) offered clear guidance for pharmacist-patient communication. This step-by-step approach framed an organized process for mastering medication counseling.

*“This model gives me a clear roadmap for talking to clients smoothly. It helps me know what I am aiming for at each step. Having a set model to follow during counseling is super helpful*,* especially for someone like me who’s new to counseling and still figuring things out.” (Control S3)*.

*“That’s where this model swoops in to save the day. It helps me piece together the whole counseling puzzle*,* giving my brain a clear guide to follow. I like it because it’s well organized*,* so I won’t accidentally skip any steps.” (Intervention S4)*.

#### Visual and auditory cues synchronized in videos complement PowerPoint slides to conceive medication counseling process effectively

Some students mentioned that videos synchronized numerous visual and auditory cues, which were challenging to convey through text or static photos typically presented by PowerPoint slides. Visual elements served as the main source of information, complemented by audio to further elaborate on the content. The enhanced visual presentation helped students better understand the counseling procedures addressed in PowerPoints slides.

*“Think about medication counseling like a conversation*,* right? When there’s stuff you can see and hear*,* it tends to stick with you longer. That’s just how we learn*,* you know?” (Control S1)*.

*“Text-only slides are tough to sneak into my brain. But with a video*,* I’ll definitely remember what the pharmacist told the patient. I’ll jot it down like…” (Intervention S2)*.

Videos attracted more students’ attention than text. However, students emphasized that solely relying on videos without accompanying PowerPoint slides lacked coherence. They suggested that video technology should be integrated alongside PowerPoint slides to provide a constructivist framework to engage learners with video-based learning more effectively.

*“The SAIDS model is great for OTC counseling*,* but how do you actually make it work? Well*,* watching videos grab everyone’s attention more than just reading text. They bring it to life with moving images and real voices. They give us way more insight than PowerPoint slides alone.” (Control S2)*.

*“When a slide is loaded with text*,* I’m just not feeling it. But for certain procedures*,* a bit of text helps. If it’s all crammed onto slides*,* I might zone out. Even if I skim through it*,* it doesn’t stick like when I see it happens in a video. That’s when it clicks*,* and I remember it better because I’ve seen it in action.” (Intervention S5)*.

#### Verbal and nonverbal signals conveyed through videos aid in the practice of medication counseling

Most students experiencing video-based learning indicated that the gesture and tone portrayed in the videos assisted their conception of how to map communication principles and theories to OTC counseling, especially regarding courtesy, empathy, and facial expressions. Moreover, they found it easy to observe pharmacists in the videos prompting questions, alternating between open-ended and closed-ended questions, and organizing the sequence of questions during counseling.

*“When watching the pharmacist look at the customer*,* it’s like he’s already knowing what I’m gonna ask next. And besides just talking about SAIDS*,* the video also shows me some slick phrases to smoothly move through each counseling step.” (Intervention S4)*.

*“It’s all about the vibes and the way people talk in the videos. You can pick up on their reactions*,* facial expressions*,* whether the customer is getting antsy or totally trusts the pharmacist. These are things that speak louder than words.” (Intervention S3)*.

#### Real-life scenario-based learning facilitates students’ ability to connect communication theory with practical applications

Students who received PowerPoint slide-based learning showed that utilizing the SAIDS flowchart offer a foundation for OTC counseling. However, they found it challenging to imagine and conceptualize how to seamlessly apply theories to practice for medication counseling with clients. Conversely, participants in video-based learning expressed that videos incorporating captions and real pharmacist-patient interactions helped them grasp how the SAIDS approach was integrated in pharmacy practice settings.

*“I feel like having real-life examples make it easier for me to get what this model is all about. Because if it’s just a model by itself*,* it’s like*,* okay*,* S*,* A*,* I*,* D*,* and S*,* got it. But with a video and a caption*,* it just clicks better for me. Seeing it in action really drives it home. Examples just hit different!” (Control S5)*.

*“Maybe I’m a bit set in my ways*,* so it’s hard for me to adapt something that’s so by-the-book. But funny enough*,* those structured principles actually help me get how counseling should work. So*,* checking out different videos could really help me see how this model play out in different scenarios.” (Intervention S1)*.

### Integration of quantitative and qualitative findings

The qualitative analysis further elucidates certain aspects of the quantitative data. Specifically, students undergoing video-based learning exhibited significant differences in their counseling performance, particularly demonstrating better performance in areas such as courtesy, nonverbal communication, and the use of diverse communication strategies, compared to those who solely received learning through PowerPoint slides. The qualitative insights suggest that videos enhance the material presented in slides by providing context and visual representations of the SAIDS model discussed in text. Through synchronized visual and auditory cues, students were provided with a model to understand how to greet clients using appropriate and structured probing sentences upon their first encounter. This authentic demonstration of OTC counseling illustrates professional skills for recognizing clients’ needs, empathizing with their emotions, and taking appropriate actions beyond verbal communication. Furthermore, the real-life case scenarios displayed by real people offered students practical guidance on approaching clients and addressing their issues across various situations, such as selecting suitable OTCs for symptom relief when students faced uncertainty. Video-based learning appears to enhance students’ ability to conceptualize the SAIDS approach and integrate it into the practice of OTC counseling in community settings.

## Discussion

The results from this mixed-methods study corroborate the application of blended multimedia learning to improve pharmacy students’ self-efficacy and performance in OTC counseling. While both cohorts of students reported enhanced self-efficacy, those exposed to a combination of slides and videos demonstrated superior counseling abilities than those who received slide-only instruction. These findings contribute to the ongoing improvement of pedagogical methods for teaching professional communication skills within pharmacy education. Integrating videos that showcase real-life patient-pharmacist interactions could serve as a valuable complement to the conventional slide-based teaching approach.

Video combines visual and auditory elements, making it a multimodal learning experience. This can enhance comprehension and retention by appealing to learners of various learning styles. Drawing on the MLT, videos tap into our cognitive infrastructure by engaging both visual and auditory systems [21]. Sweller and Bandura highlighted the use of symbolic models through words and images to disseminate knowledge effectively to large audiences [44]. In this study, students expressed that videos aided in conceptualizing how to counsel and deliver information that may be challenging to convey through text or static image alone. Visual elements function as the primary source of information, while audios elaborate on the information. Previous research also demonstrated that when audio and visual materials were co-presented, knowledge retention tended to increase more significantly compared to using either visual or audio presentation alone [45]. Therefore, integrating videos into learning environments can potentially lead to improved learning outcomes, as evidenced by the enhanced counseling performance observed among students who learned through videos in this study.

Videos allow authentic demonstration of skills, procedures, and exercises with real people for OTC counseling. This can especially benefit learners who acquire professional skills through observation and mimicry. Students indicated the SAIDS approach provides them an efficient framework for engaging with clients requiring OTCs. However, they noted that text-heavy information used to introduce the SAIDS approach is difficult to comprehend or apply to real-life scenarios. Pre-recorded videos serve to demonstrate communication principles discussed in slides, contextualizing abstract concepts that may be challenging to convey through text alone. Students can perceive how communication skills are carried out in the pharmacy routine through their eyes and ears. For instance, showing proper gestures and body language to convey empathy and respect is more effectively conveyed through video format than written instructions. In particular, learners tend to adopt a cognitive approach in which knowledge is generated through stepwise learning in video lectures [46]. Video technology alongside PowerPoint slides foster a sequential framework to engage learners to learn with videos [46]. Students could apply their gained knowledge in practice by identifying similarities and discrepancies between communication theories and case scenarios after watching the video. Consequently, it is not surprising that students who received video-based learning performed better in demonstrating courtesy and nonverbal communication than those who relied solely on slide-based lectures. Integration of videos complements information presented with text, sounds, and images to create multimedia learning environment may lead to better learning outcomes. Meta-analyses of video effects on higher education learning confirmed that adding video to existing teaching methods yields stronger learning improvements than replacing existing teaching methods with videos [19]. Our study findings resonate with the existing knowledge, showing that students exceled in medication counseling and expressed more benefits from integrating videos into slide-based lectures.

Video content tends to be more engaging than text-based content, which can help hold viewers’ attention and improve information retention. This is particularly important for health education, aiming to promote behavior change. Studies indicate that videos featuring real people performing the desired behaviors are more effective to engage audience in behavior change than those solely presenting verbal or graphical information [47]. Modeling examples offers students the opportunity to observe an adult or a peer model performing the given tasks. This study adapted real-life cases portrayed by a practicing pharmacist, enabling students to relate to similar pharmacy backgrounds and envision their future practice [48]. Each video case was kept short (less than 5 min) for maximizing student attention and mimicking diverse routine scenarios in community pharmacies [49]. As a result, students may exhibit greater commitment and motivation to engage in behavioral rehearsals that may happen in future practice. Video technology allows students to learn medication counseling by observing or imitating the pharmacist’s actions, words, or expressions [44]. The Social Learning Theory and Cognitive Load Theory both emphasize learning through observation and imitation [16]. They also highlight the efficiency of learning from others’ experiences, rather than merely through personal experimentation [16, 50, 51]. When modeling examples include instructional explanations, learners are more likely to benefit more as their attention is drawn to both the model’s speaking and behaviors. Incorporating instructional explanations into video-based modeling has been shown to yield better results compared to merely observing the modeling alone [44]. In this study, the instructor provided explanation and facilitated discussion after each video segment to enhance students’ metacognition via linking theory to self-reflection and practice execution. Additionally, providing positive examples had a beneficial impact on students’ ability to apply learned concepts to practical tasks [44]. This echoes Baldwin’s discovery that learning is enhanced when there is similarity in stimuli and repetition throughout the learning process [52]. Students can connect their background knowledge acquired from slides to the scenarios depicted in videos, thus promoting the reproduction of behaviors.

### Study strengths and limitations

The study was conducted during the academic years 2021–2022 and 2022–2023. Although the COVID-19 pandemic was ongoing during the early stages of this period, it did not significantly affect the implementation of the study program, as institutional operations and participant engagement had largely returned to normal operations. The instructional design incorporated multimedia learning and authentic role-playing to teach and assess pharmacy students’ competence in OTC counseling. Multimedia learning helped connect text-heavy content with real-life applications, while authentic role-playing served as an effective method for assessing counseling performance [53]. Evaluation methods focused on attitudes and behaviors, targeting higher-order outcomes such as learning gains and behavioral changes, as outlined in Kirkpatrick’s four-level evaluation model [54]. A mixed methods approach was used to integrate quantitative and qualitative data, providing deeper insight into the potential mechanisms through which video-based learning enhanced counseling performance, beyond statistical significance.

However, the study had several limitations. Participants were recruited from a single school of pharmacy, and the use of convenience sampling with a relatively small sample size may limit the generalizability of the findings. Future research could explore the implementation of the demonstrated pedagogical approaches on a larger scale to better assess their impact on students’ OTC counseling skills. Moreover, the study did not evaluate the effects of the intervention in real-world practice, as student performance was assessed only through simulated patient interactions within the academic setting. The evaluation was also based on a single case scenario relevant to community settings. Future studies should investigate the effectiveness of the SAIDS approach across a range of case scenarios and in real-world community settings involving actual clients.

## Conclusions

Multimedia instruction, especially video-based learnings, enhance pharmacy students’ performance in OTC counselling. While their self-efficacy also improved, the increase was comparable to that seen with traditional slide-based teaching. To further bolster pharmacy students’ competence in this domain, it is advisable to integrate video-based instruction with traditional lecture-based approaches.

## Figures and Tables

**Figure:**
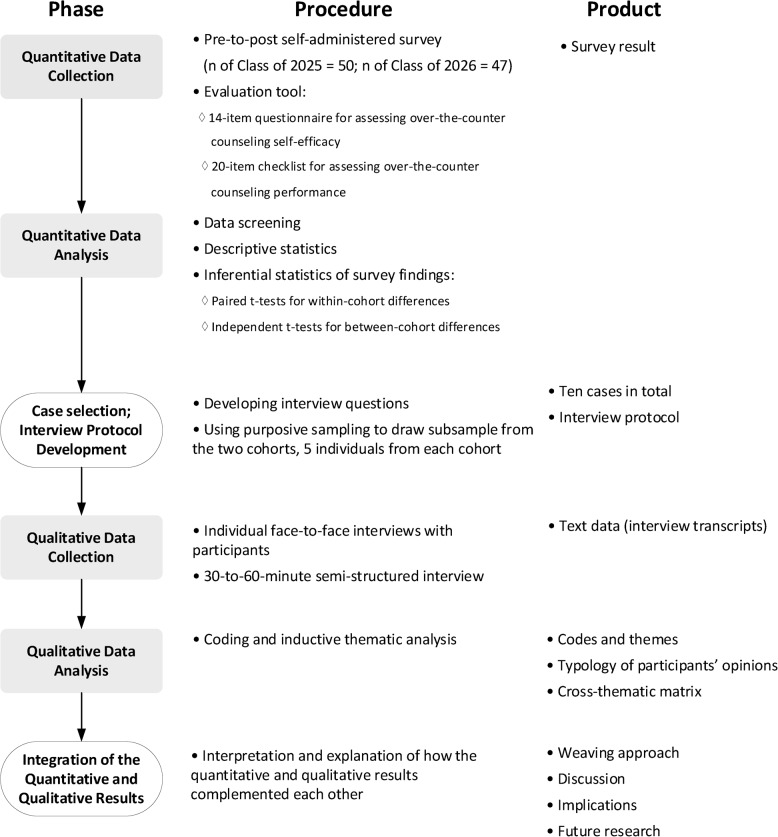
Explanatory sequential mixed methods design for exploring impacts of multimedia pedagogy on pharmacy students’ medication counseling competence

## Data Availability

The study materials and the detail of all analyses are available from the corresponding author upon reasonable request.

## Appendix 2

**Table:** A checklist for evaluating pharmacy students’ communication skills for over-the-counter medication counseling

Item	Question
1.	Greet the patient and introduce yourself.
2.	Identify whether the patient has time needed for the counseling.
3.	Identify if with any allergy.
4.	Identify existing prescription medications taken.
5.	Identify other over-the-counter (OTC) medications taken.
6.	Identify other nutritional supplements taken.
7.	Identify co-morbid conditions.
8.	Assess if the patient is an appropriate self-care candidate.
9.	Recommend appropriate OTC product for self-care.
10.	Counsel on the indication of the recommended OTC product(s).
11.	Counsel on when a benefit from the specific OTC product could be perceived.
12.	Counsel on how to take the specific OTC product.
13.	Counsel on common adverse effects/precautions of the OTC product.
14.	Counsel on strategies for coping with common adverse effects/precautions of the OTC product.
15.	Answer the patient’s questions if any.
16.	Assess that the patient understands information.
17.	Provide a structured and friendly ending.
18.	Nonverbal cues appropriate/helpful.
19.	Provide an empathic response.
20.	Use tools to facilitate effective OTC counseling.

## Appendix 3

**Table:** Questions used in the semi-structured interview

1	What scenario would be ideal for effective delivery of medication counseling with clients?
(1)	What are the essential elements for efficient medication counseling?
2	When providing medication counseling to a client, which communication skills do you find easiest to apply? How do these skills influence the way you communicate with clients?
3	When providing medication counseling to a client, which communication skills do you find most difficult to use? How do these challenges affect your communication with clients?
4	How does the SAIDS counseling approach assist you in providing medication counseling to the public? Are there any communication skills not addressed by this approach?
5	What changes would you suggest to the structure of the communication course to further enhance students’ skills in medication counseling?

## Abbreviations

ADR: Adverse drug reaction
MLT: Multimedia Learning Theory
OTC: Over-the-counter
SAIDS: Self-Assessment-Informed Decision-making-System
SCT: Social Cognitive Theory
